# Cincinnati Correspondence

**Published:** 1868-06-15

**Authors:** 


Cincinnati, June 1st, 1868.

My Dear Doctor:—The profession of this city are not idle, even if unusual health prevails. The activity of our Faculty, intellectually, will compare favorably with that of any portion of the West. This can in no way be better attested than by witnessing the proceedings of the Academy of Medicine, at any one of its weekly meetings. Recently an original paper was read by the President of the Academy, I. Davis, M.D., on diphtheria. Dr. Davis took the position that this disease was "epidemic sore throat," and that epi demic sore throat was diphtheria. In other words, that the terms are antistrophic.

Prof. Richardson represented the evil tendency of such a position, syllogistically. Thus — Mrs. Smith's baby has a sore throat. The baby gets well, after taking the doctor's medicine a few days, without any serious illness.

The conclusion is that the doctor has cured a case of diphtheria. The injury to the profession from this style of argument, results from the fact that most of them have taught that this disease was infrequent, and the mortality great. The discussion lasted several months, but the *points* are as stated above.

This was followed by the introduction of another matter of great importance, by Prof. James Graham, who urged the moral aspect of the question with much eloquence (would that other members of the profession entertained such sentiments of high morality!) The following, in accordance with his views, at the regular meeting, May 25th, 1868, was unanimously adopted by the special committee appointed to report on "procured abortions and criminal advertisements":

"The committee, to whom was referred the consideration of procured abortions, and the criminal advertising of the means and instrumentalities of producing the same, or preventing impregnation, respectfully report as follows:

"1. Criminal abortions are fearfully frequent.

"2. That as a general rule, the crime is committed by irregular practitioners of medicine, by certain female *accoucheurs*, and by apothecaries, who vend certain nostrums to correct suppressed menstruation.

"3. That we believe that the advertisement of abortionists and abortion drugs encourages the practice of abortion, and is criminal, and, therefore, the Academy of Medicine should earnestly protest against the admission by the press of such advertisements.

"4. That the Academy appoint a standing committee to fortify, as much as possible, the Health Officer in Cincinnati, in the prosecution of such offenders.

"6. That it is the duty of all good citizens, and especially physicians, to discourage the circulation and patronage of the journals in which are published the advertisements of those who profess to produce abortion, or prevent impregnation.

"J. F. WHITE, M.D.,
"JAMES GRAHAM, M.D.,
"F. G. SCHMIDT, M.D.,
"C. S. MUSCROFT, M.D.,
"JOHN DAVIS, M.D., *President*,
"JOHN L. NEILSON, M.D., *Rec. Sec'y*.

"After the report had received the signatures of the members of the Academy, Drs. James Graham, George Mendenhall, and A. M. Johnson were appointed a standing committee to carry out its provisions."

Prof. C. G. Comegys offered a minority report, which, after being read, was not received by the Academy, consequently I am unable to furnish you with a copy.

From the well known character of the gentlemen appointed on the standing committee, to give back bone to the matter, it is expected that there will be something accomplished to remedy this growing evil. ***

